# Metabolomics and lipidomics of plasma biomarkers for tuberculosis diagnostics using UHPLC-HRMS

**DOI:** 10.3389/fcimb.2025.1526740

**Published:** 2025-06-30

**Authors:** Gaofeng Sun, Quan Wang, Xinjie Shan, Maierheba Kuerbanjiang, Ruiying Ma, Wensi Zhou, Lin Sun, Qifeng Li

**Affiliations:** ^1^ Graduate of School, Xinjiang Medical University, Urumqi, China; ^2^ Department of Medical Laboratory, The Infectious Disease Hospital of Xinjiang Uygur Autonomous Region, Urumqi, China; ^3^ Department of Medical Laboratory, Xinjiang Institute of Pediatrics, Xinjiang Hospital of Beijing Children’s Hospital Children’s Hospital of Xinjiang Uygur Autonomous Region, The Seventh People’s Hospital of Xinjiang Uygur Autonomous Region, Urumqi, China; ^4^ Laboratory of Respiratory Diseases, Beijing Pediatric Research Institute, Beijing Children’s Hospital, Capital Medical University, Beijing Key Laboratory of Core Technologies for the Prevention and Treatment of Emerging Infectious Diseases in Children, Key Laboratory of Major Diseases in Children, Ministry of Education, National Clinical Research Center for Respiratory Diseases, National Center for Children’s Health, Beijing, China; ^5^ Department of Science and Education, Xinjiang Institute of Pediatrics, Xinjiang Hospital of Beijing Children’s Hospital, Children’s Hospital of Xinjiang Uygur Autonomous Region, The Seventh People’s Hospital of Xinjiang Uygur Autonomous Region, Urumqi, China

**Keywords:** Tuberculosis, metabolite, UHPLC-HRMS, diagnosis, biomarker, machine learning

## Abstract

**Introduction:**

Determining metabolic profiles during host-pathogen interactions is crucial for developing novel diagnostic tests and exploring the mechanisms underlying infectious diseases. However, the characteristics of the circulating metabolites and their functions after *Mycobacterium tuberculosis* infection have not been fully elucidated. Therefore, this study aimed to identify the differential metabolites in tuberculosis (TB) patients and explore the diagnostic value of these metabolites as potential biomarkers.

**Methods:**

Seventy-two TB patients and 78 healthy controls (HCs) were recruited as the training set, while 30 TB patients and 30 HCs were enrolled as the independent validation set. Metabolites in plasma samples were analyzed by high-resolution mass spectrometry. Differential metabolites were screened using principal component analysis and machine learning algorithms including LASSO, Random Forest, and XGBoost. The diagnostic accuracy of the core differential metabolites was evaluated. Pearson correlation analysis was performed.

**Result:**

The metabolic profiling of TB patients showed significant separation from that of the HCs. In the training set, 282 metabolites were identified as differentially expressed in TB patients, with 214 metabolites validated in the independent validation cohort. KEGG pathway enrichment analysis showed that the differential metabolites were mainly enriched in lipid metabolism. Seven core differential metabolites were identified by the three machine learning algorithms. Receiver operating characteristic analysis revealed that Angiotensin IV had high accuracy in diagnosing TB.

**Conclusion:**

These newly identified plasma metabolites are expected to serve as potentially valuable biomarkers for TB, potentially facilitating the diagnosis of the disease and enhancing the understanding of its underlying mechanisms.

## Introduction

Tuberculosis (TB) is an ancient and serious global infectious disease caused by *Mycobacterium tuberculosis* (MTB). Despite significant advancements in management and prevention strategies, accurate disease diagnosis and progression monitoring still face many challenges (World Health Organization, 2024), which heavily block the global targets to reduce mortality from active TB. Therefore, the development of novel diagnostic methods is urgently needed.

The progression of TB can vary depending on the interplay between the host and the bacterium. MTB infection triggers a series of complex immune responses in the host (Ndong et al., 2022), involving not only the activation and regulation of immune cells but also alterations in the host’s metabolic state (Vrieling et al., 2018). The interaction between the host cells and MTB affects various metabolic pathways, including energy metabolism, lipid metabolism, and amino acid metabolism (Rameshwaram et al., 2018; Laval et al., 2021). These metabolic changes may exhibit different characteristics in different stages of TB (such as the latent and active period) and may vary due to individual genetic backgrounds, nutritional statuses, and environmental factors (Singh et al., 2009).

Currently, the diagnostic methods for TB mainly include traditional bacteriological tests (such as smear microscopy and sputum culture), immunological tests (such as tuberculin skin test and gamma-interferon release assays), and recently widely used molecular diagnostic techniques (such as GeneXpert) (Boloko et al., 2022). However, bacteriological tests are limited in their capacity for rapid clinical diagnosis due to low sensitivity and time-consuming procedures. Immunological tests face challenges in distinguishing between latent and active TB. Furthermore, current molecular diagnostic techniques reliant on respiratory tract specimens encounter challenges in widespread application for patients who produce little or no sputum, such as children. Therefore, detecting biomarkers in plasma represents a valuable supplementary method for disease diagnosis (Captur et al., 2020; Chu et al., 2019).

As the ultimate downstream pool of genome transcription, metabolites directly reflect the events occurring during the host-pathogen interactions. In recent years, metabolomics has been widely used in the study of both infectious diseases (Li et al., 2022; Song et al., 2021; Zardini Buzatto et al., 2020) and non-infectious diseases (Cheng et al., 2025; Valo et al., 2025). However, studies on the potential metabolites associated with TB and their underlying mechanisms in TB progression are relatively rare. The application of ultra-high-performance liquid chromatography coupled with high-resolution mass spectrometry (UHPLC-HRMS) offers considerable advantages in exploring the metabolic biosignatures of TB due to its high sensitivity, selectivity, and excellent reproducibility in time retention (Hartling et al., 2021). Therefore, we aimed to screen differential metabolites in TB patients and analyze their potential as biomarkers for diagnosis. Multivariate statistical analysis and machine learning algorithms (Yao et al., 2024) were then performed to identify the core metabolites with significant diagnostic value. Our findings revealed the metabolic characteristics of TB and may provide evidence for improving the diagnostic efficiency and accuracy of the disease.

## Materials and methods

### Study participants

Patients with pulmonary TB aged 18-60 years were recruited from the Infectious Diseases Hospital of Xinjiang Uygur Autonomous Region from January to March 2024. They were diagnosed according to the diagnostic guidelines of China (Sun et al., 2023). Inclusion criteria: 1) patients with positive etiological and pathological results; 2) patients with at least one TB-related symptom or sign; 3) patients with radiographic evidence consistent with TB; and 4) patients with positive results from tuberculin skin test or gamma-interferon release assays. Exclusion criteria: 1) patients with comorbidities such as diabetes mellitus, autoimmune diseases, pulmonary tumors, or other pulmonary infections; 2) patients who had received immunomodulatory agents or anti-TB treatment. Concurrently, adult staff members who underwent health examinations at the Children’s Hospital of the Xinjiang Uygur Autonomous Region were recruited as healthy controls (HCs). All healthy volunteers were confirmed to be free of MTB infection, lung diseases, immune deficiencies, or immunosuppression.

In total, 72 patients with active TB and 78 HCs were recruited as the training set. The independent validation cohort included 30 TB patients and 30 HCs. The demographic characteristics of these participants are presented in **Table 1**. This study was approved by the Ethics Committee of the Children’s Hospital of Xinjiang Uygur Autonomous Region (Approval No. KY2024083006) and all methods were performed following the relevant guidelines and regulations under the committee’s supervision.

**Table 1:** Demographic characteristics of the participants.

Characteristics	Training set (n=150)		Independent testing set (n=60)	
	TB	HC	TB	HC
Sample size	72	78	30	30
Gender(male/female)	37/35	9/69	14/16	6/24
Age(years)^a^	64.0(55.0,71.0)	34.0 (28.8,40.3)	63.5 (50.0,70.8)	39.0 (32.5,44.3)
Severity of TB (severe/non-severe)	5/67	/	2/28	/

TB, tuberculosis; HC, healthy control. ^a^Data are presented as mean (interquartile range).

### Sample preparation

Whole blood was collected into EDTA tubes and centrifuged at 3000 g for 10 min to obtain the plasma. The plasma (100 μL) was mixed with 400 μL of 80% methanol aqueous solution. The mixture was incubated for 5 minutes on ice and then centrifugated at 15000 g at 4°C for 20 minutes. The supernatant was transferred to a new centrifuge tube and diluted to achieve a methanol content of 53%. After centrifugation again at 15000 g at 4°C for 20 minutes, the supernatant was analyzed with UHPLC-HRMS.

### UHPLC-HRMS

UHPLC-HRMS analyses were performed by Novogene Co., Ltd. (Beijing, China). A Vanquish UHPLC system (Thermo Fisher, Dreieich, Germany) coupled with a Q Exactive™ HF mass spectrometer (Thermo Fisher, Dreieich, Germany) was used. Samples were injected into a Hypesil Goldcolumn (100×2.1 mm, 1.9 μm, Thermo Fisher, Dreieich, Germany) at a flow rate of 0.2 mL/min. The column temperature was set at 40°C. Elution gradient was performed using a binary solvent system consisting of 0.1% formic acid in water (solvent A) and methyl alcohol (solvent B). The solvent gradient was set as follows: 2% B, 1.5 min; 2-85% B, 3 min; 85-100% B, 10 min;100-2% B, 10.1 min; 2% B, 12 min. The Q Exactive™ HF mass spectrometer was operated in positive and negative polarity modes, with the following parameters: scan range: 100-1500 *m/z*; spray voltage: 3.5 kV; sheath gas flow rate: 35 psi; auxiliary gas flow rate: 10 L/min; capillary temperature: 320°C; S-lens RF level: 60; auxiliary gas heater temperature: 350°C. Samples were injected with a volume of 2 µl and analyzed with a total run time of 12 min. Accurate mass spectra were acquired using data-dependent scans in both polarity modes. A polarity switching was used for electrospray ionization. The Q Exactive HF was calibrated before every batch analysis and the quality control (QC) pooled samples were used as in previous studies (Sun et al., 2023). The positive and negative mass calibration was performed using Thermo Scientific™ Pierce™ Negative Ion Calibration Solution and Thermo Scientific™ Pierce™ LTQ ESI Positive Ion Calibration Solution (Thermo Fisher, Dreieich, Germany), respectively.

### Data processing

The raw data files generated by UHPLC-HRMS were processed using Compound Discoverer 3.3 (CD3.3, ThermoFisher). The peak alignment, peak picking, and quantitation for each metabolite were analyzed. The main parameters were set as follows: the peak area was corrected with the first quality control sample, with an actual mass tolerance of 5 ppm, a signal intensity tolerance of 30%, and a minimum intensity threshold. After that, peak intensities were normalized to the total spectral intensity. The normalized data was used to predict the molecular formula based on additive ions, molecular ion peaks, and fragment ions. Peaks were then matched with mzCloud (https://www.mzcloud.org/), mzVault, and the MassList database to obtain accurate qualitative and relative quantitative results. Data processing was performed using the Linux system (CentOS version 6.6), statistical software R (R version R-4.2.3), and Python (Python 2.7.6 version). These metabolites were annotated using the KEGG database (https://www.genome.jp/kegg/pathway.html), HMDB database (https://hmdb.ca/metabolites), and LIPID MAPS database (http://www.lipidmaps.org/).

### Statistical analysis

Principal component analysis (PCA) was performed to evaluate the data quality of lipid metabolites in terms of homogeneity and reproducibility. Differential metabolites were defined as those having a *P*-value threshold of < 0.05 and a fold change (FC) ≥ 2 or FC ≤ 0.5 and they were visualized using Volcano plots. KEGG PATHWAY Database (http://www.genome.jp/kegg/) was used for metabolic pathways analysis. Differential metabolites were further screened using Least Absolute Shrinkage and Selection Operator (LASSO) regression analyses, Random Forest, and XGBoost algorithms in R. Pearson correlation was used for correlation analysis. Receiver operating characteristic (ROC) curve analysis was performed to evaluate the diagnostic performance of potential biomarkers. The significance level was set at P < 0.05 for all tests.

## Results

### Analysis of differential metabolites in plasma

**Figure 1:**
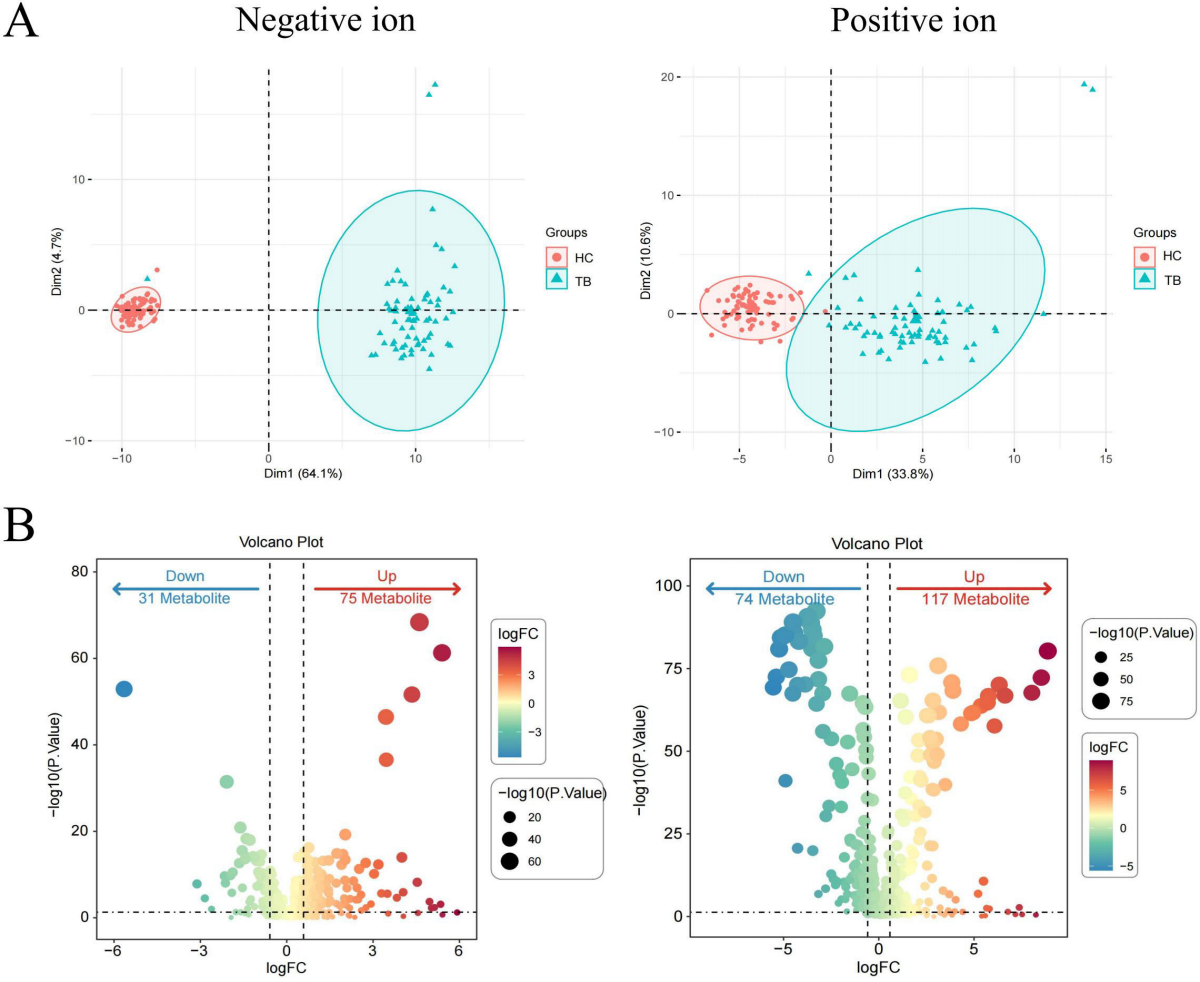
Identification of differential metabolites in TB patients in the training set. PCA plots of metabolites in the negative ion and positive ion modes **(A)**. Volcano plots of differential metabolites in the negative ion and positive ion modes **(B)**. Differential metabolites were defined by a threshold of Pvalue < 0.05 and fold change (FC) ≥ 2 or FC ≤ 0.5.

Among the 150 patients in the training set, the PCA revealed that metabolites from the same group clustered together and that there was significant separation between the two groups (**Figure 1A**). As presented in Volcano plots, there were 282 differential metabolites between TB patients and HCs (**Figure 1B**), including 31 downregulated and 75 upregulated metabolites in negative ion mode, as well as 74 downregulated and 117 upregulated metabolites in positive ion mode.

**Figure 2:**
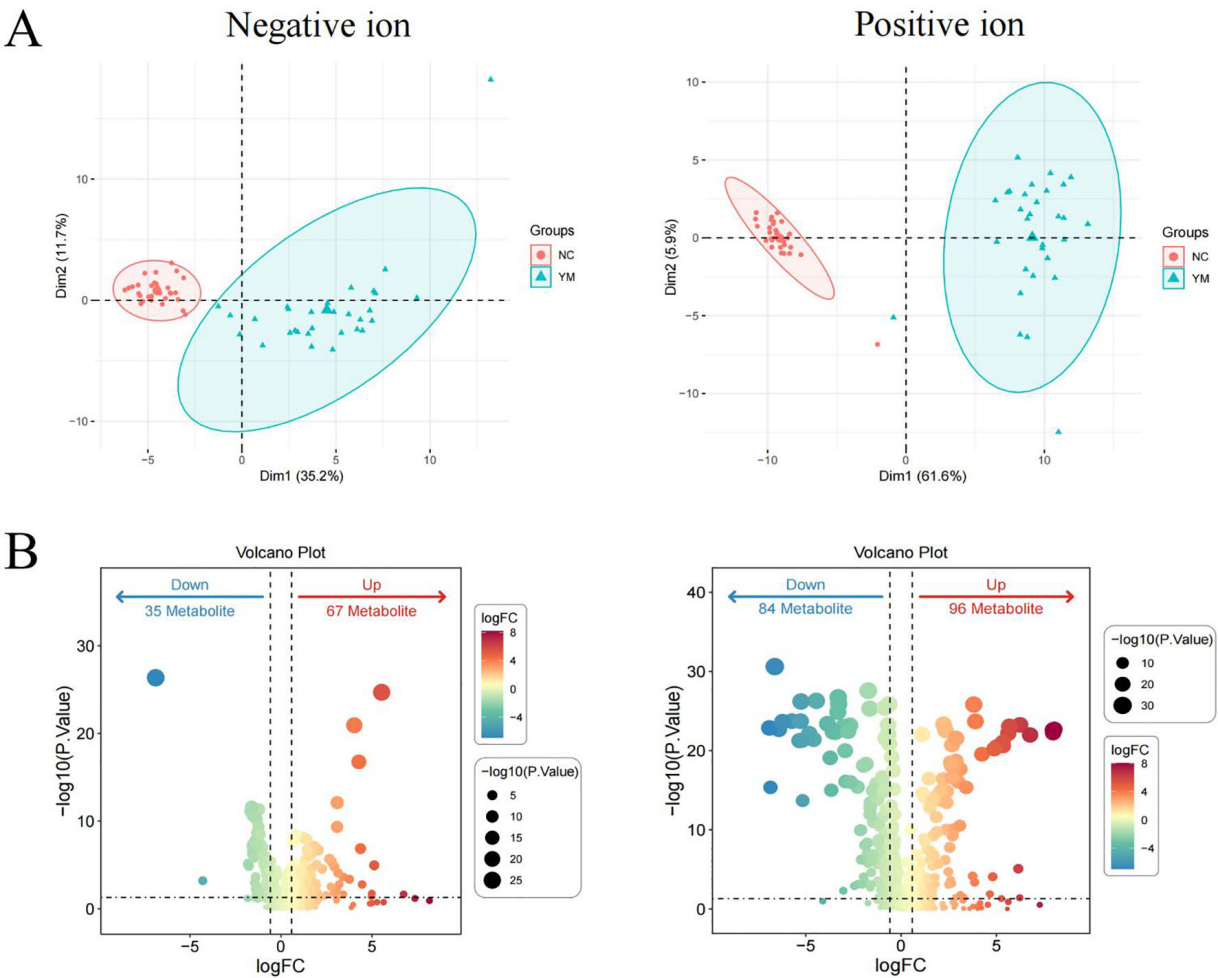
Validation of differential metabolites in TB patients in the validation set. PCA plots of metabolites in the negative ion and positive ion modes **(A)** Volcano plots of differential metabolites in the negative ion and positive ion modes **(B)** Differential metabolites were defined by a threshold of P value < 0.05 and FC > 2 or FC ≤ 0.5.

To evaluate the reliability of the differential metabolites, an independent cohort comprising 60 individuals (including 30 TB cases and 30 HCs) was used for validation. A significant separation was also observed between TB patients and HCs (**Figure 2A**). Moreover, 297 differential metabolites were identified, including 35 downregulated and 67 upregulated metabolites in negative ion mode, along with 84 downregulated and 96 upregulated metabolites in positive ion mode (**Figure 2B**).

### TB-associated differential metabolites enriched in lipid metabolism

**Figure 3:**
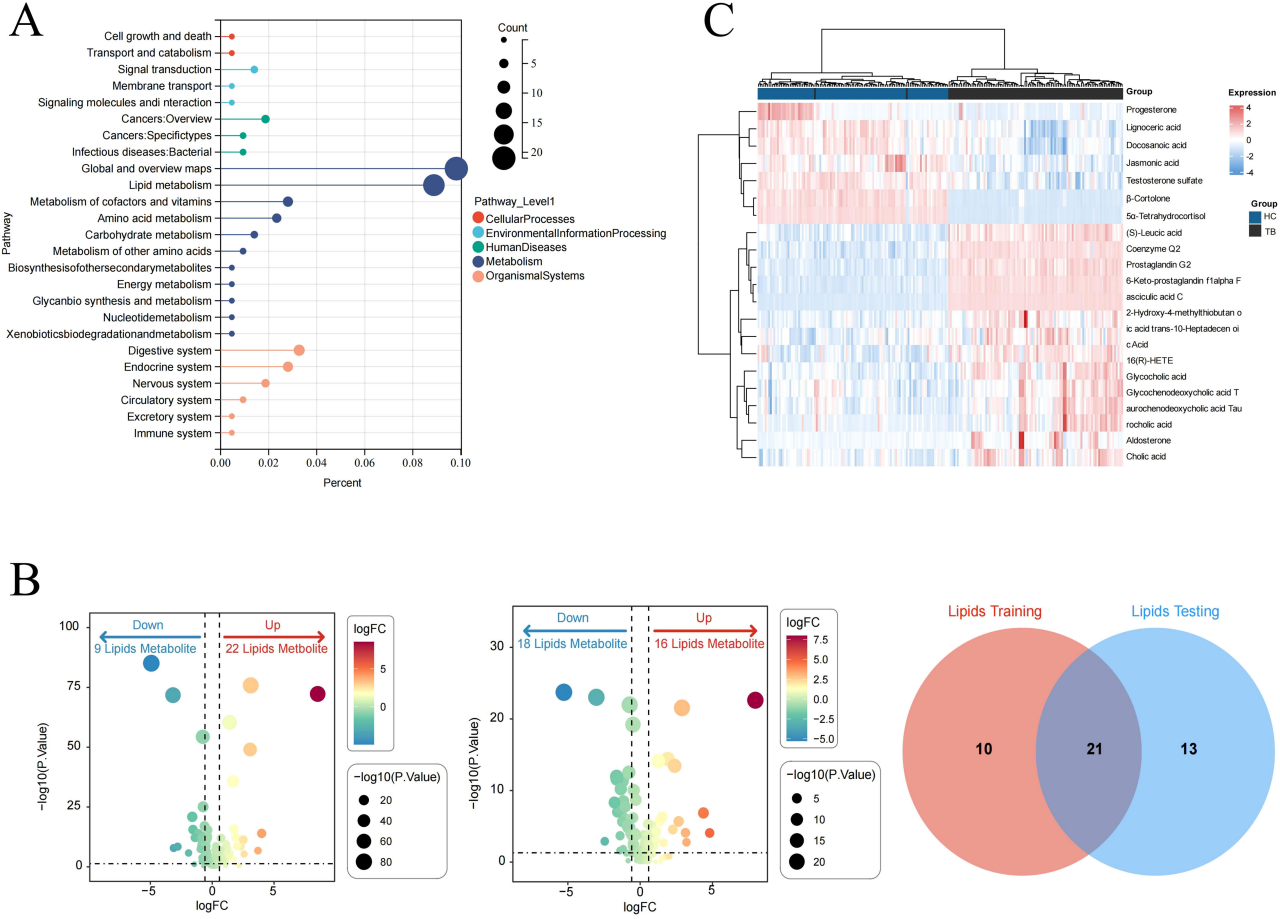
The signatures of the overlapping differential metabolites in TB. **(A)** Venn diagram of overlapping differential metabolites in the negative ion mode from the training and validation sets. **(B)** Heatmap of the differential metabolites in TB patients and HCs. **(C)** Pathway enrichment analysis of differential metabolites.

**Figure 4:**
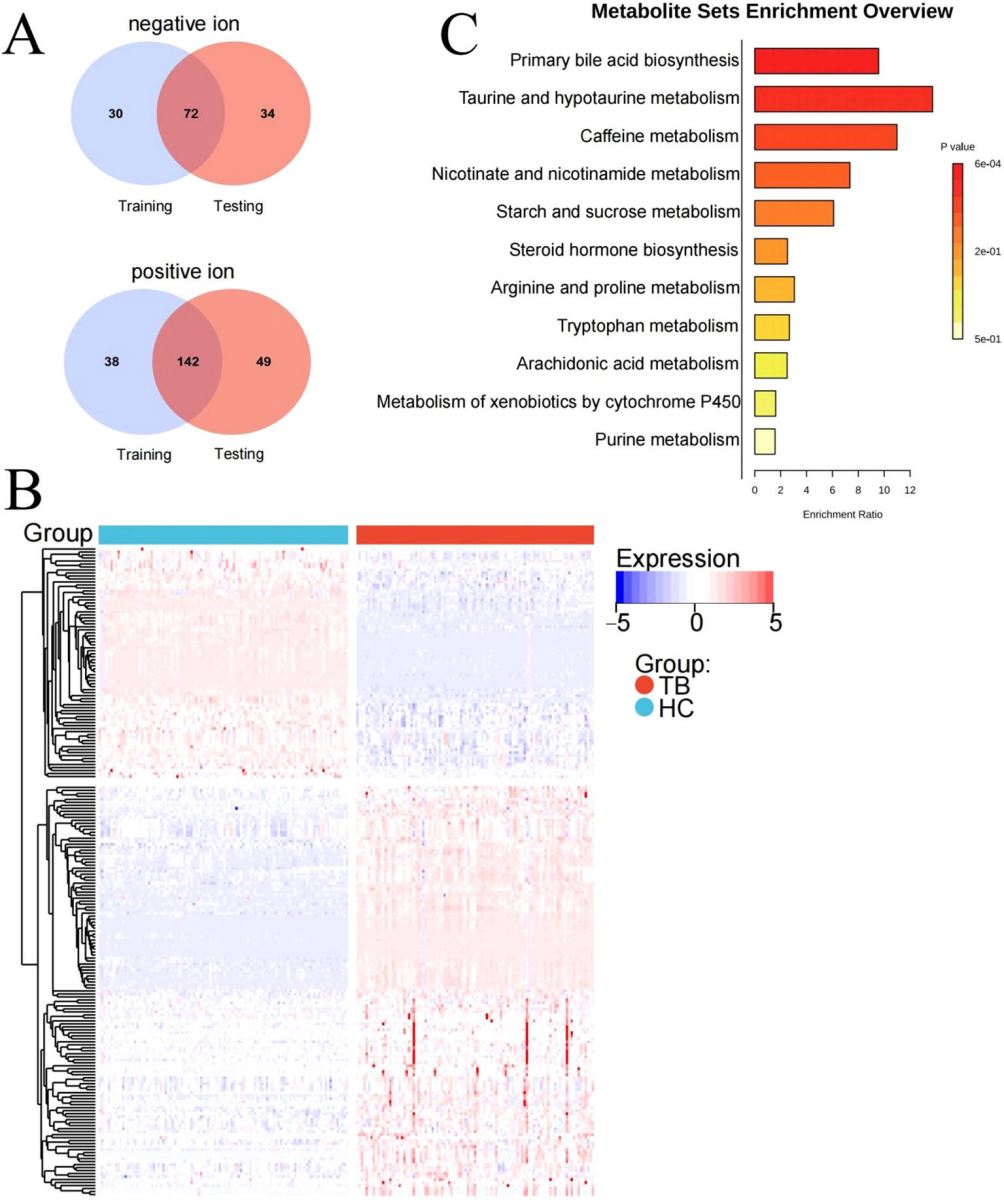
Pathway analysis of lipids dysregulated in TB patients. **(A)** KEGG pathway analysis. **(B)** Identifying the differential lipids from the training and validation sets. **(C)** Heatmap of the overlapping lipids in TB patients and HCs.

After intersecting the differential metabolites from the training and validation sets, 72 and 142 overlapping differential metabolites were identified in negative and positive modes, respectively (**Figure 3A**). The variation of the differential metabolites was visualized using a heatmap (**Figure 3B**). KEGG pathway enrichment analysis indicated that the differential metabolites were primarily enriched in the primary bile acid biosynthesis, taurine and hypotaurine metabolism, etc. (**Figure 3C**). They were also enriched in cellular processes, environmental information processing, human disease, metabolism, and the organism system. Among them, 157 differential metabolites were mainly enriched in 25 pathways, including global and overview maps, lipid metabolism, and amino acid metabolism, among others (**Figure 4A**). Furthermore, 31 and 34 differential lipids between TB patients and HCs were identified in the training and testing sets, respectively. Among them, 21 overlapping lipids showed distinct dysregulation between TB patients and HCs (**Figure 4B**). The heatmap for the 15 upregulated and seven downregulated lipids in TB patients is shown in **Figure 4C**.

### Screening of core metabolites from overlapping differential metabolites

**Figure 5:**
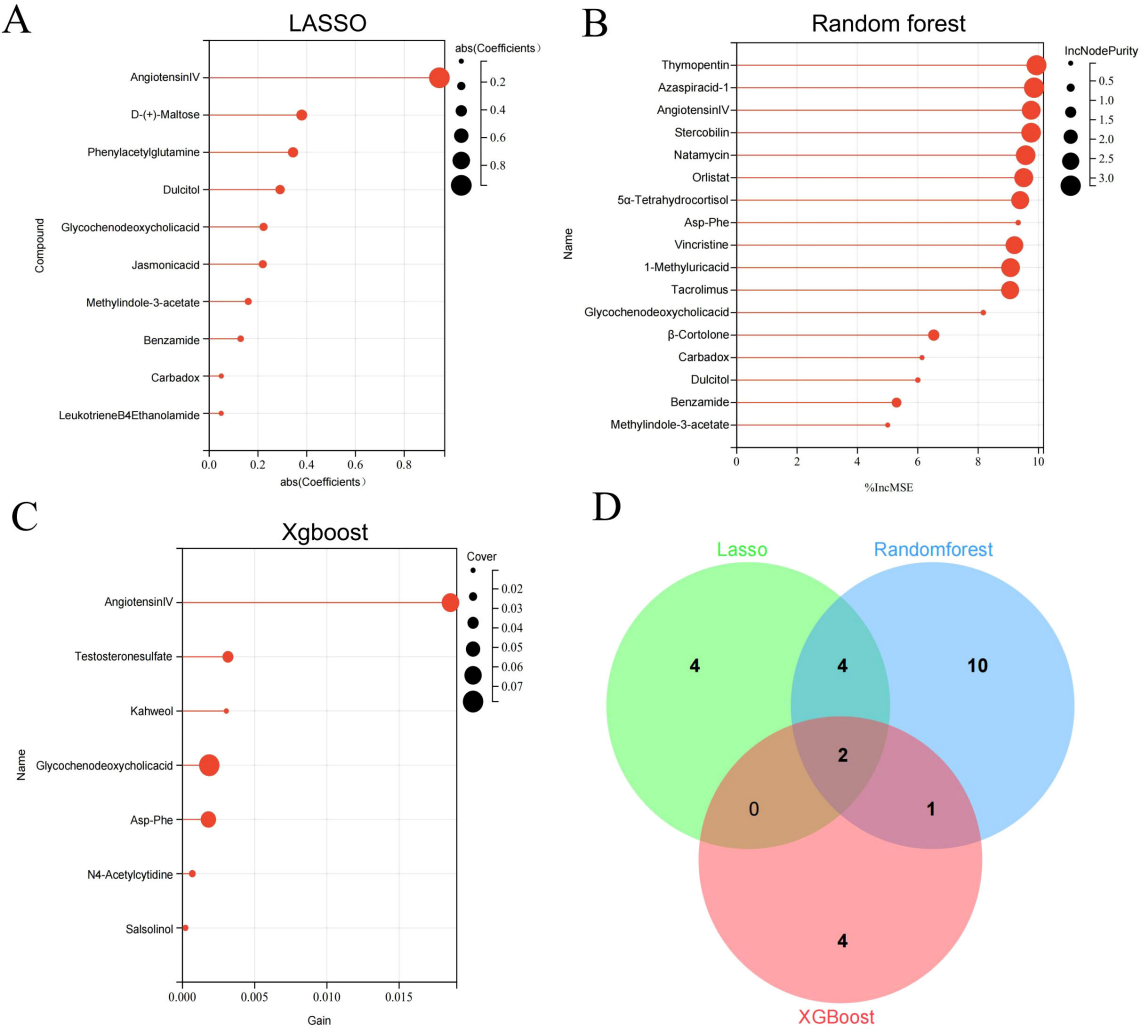
Key differential metabolites identified by three machine learning algorithms. Differential metabolites were selected by LASSO **(A)**, Random Forest **(B)**, and Xgboost **(C)**. Venn diagram of differential metabolites identified by three machine learning algorithms **(D)**.

The differential metabolites were further selected using LASSO, random forest, and XGBoost. Seven core differential metabolites were identified by at least two machine learning algorithms, including Angiotensin IV, glycochenodeoxycholic acid, methyl indole-3-acetate, dulcitol, Asp-Phe, benzamide, and carbadox. Detailed information on these seven metabolites is shown in the **Supplementary Table S1**. Finally, Angiotensin IV and glycochenodeoxycholic acid were selected by all three algorithms (**Figure 5**).

**Figure 6:**
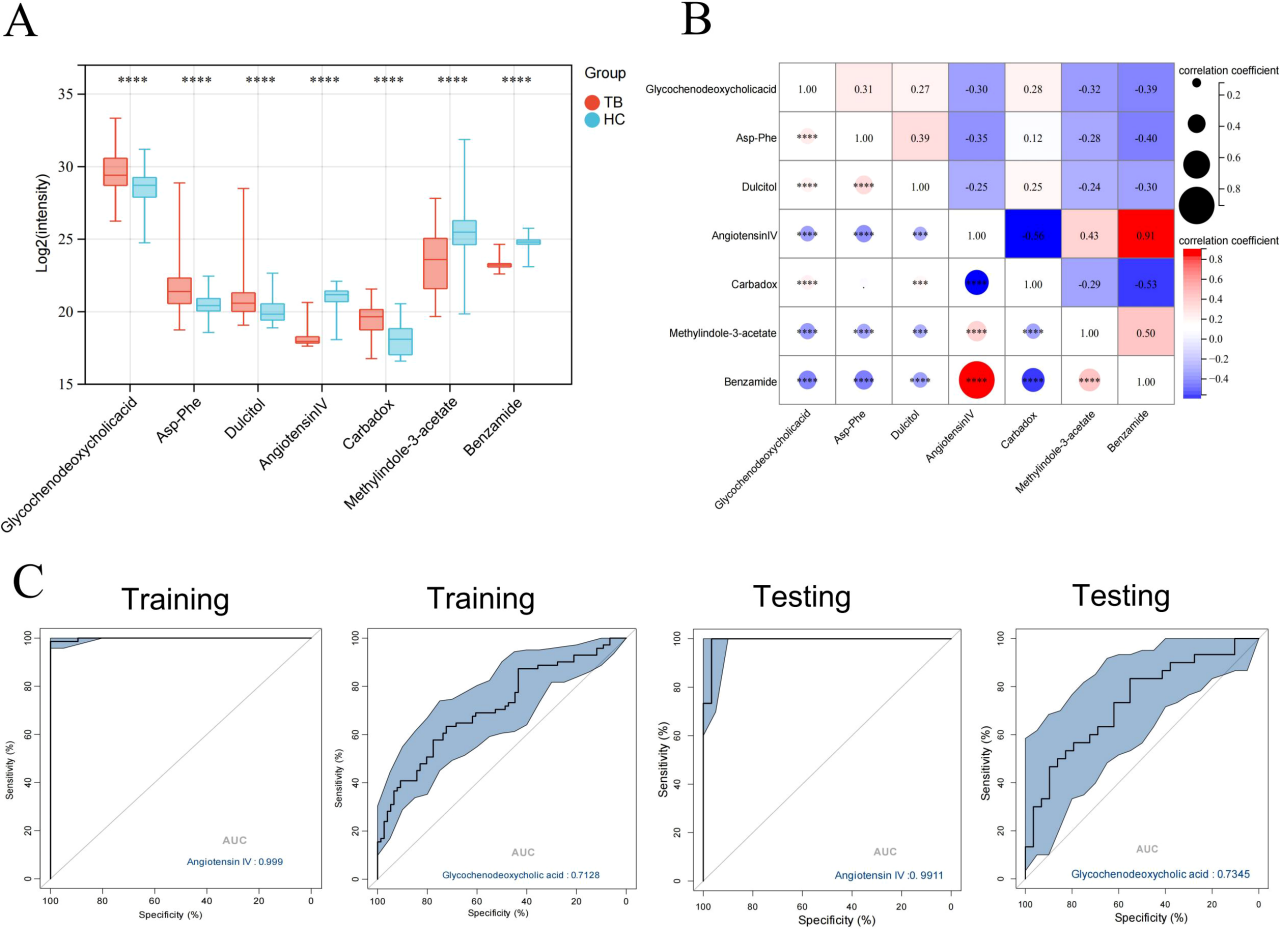
Identification of the biomarkers with potential diagnostic values. **(A)** Boxplot of core differential metabolites in the training and validation set. **(B)** Correlation plot of core differential metabolites. **(C)** ROC curve analysis of Angiotensin IV in differentiating between TB patients and HCs. *** P < 0.001,****P < 0.0001.

The seven core differential metabolites were significantly different between TB patients and HCs (**Figure 6A**), with four metabolites increased and three decreased in TB patients. Moreover, there were significant correlations among the core differential metabolites (P < 0.05) (**Figure 6B**). Additionally, ROC curve analysis revealed that Angiotensin IV had high accuracy in the diagnosis of TB (AUC = 0.9990 and 0.9911 in the training and validation sets, respectively) (**Figure 6C**). The sensitivity and specificity were 98.6% and 100.0% in the training set, and 100.0% and 96.7% in the validation set, respectively.

**Figure 7:**
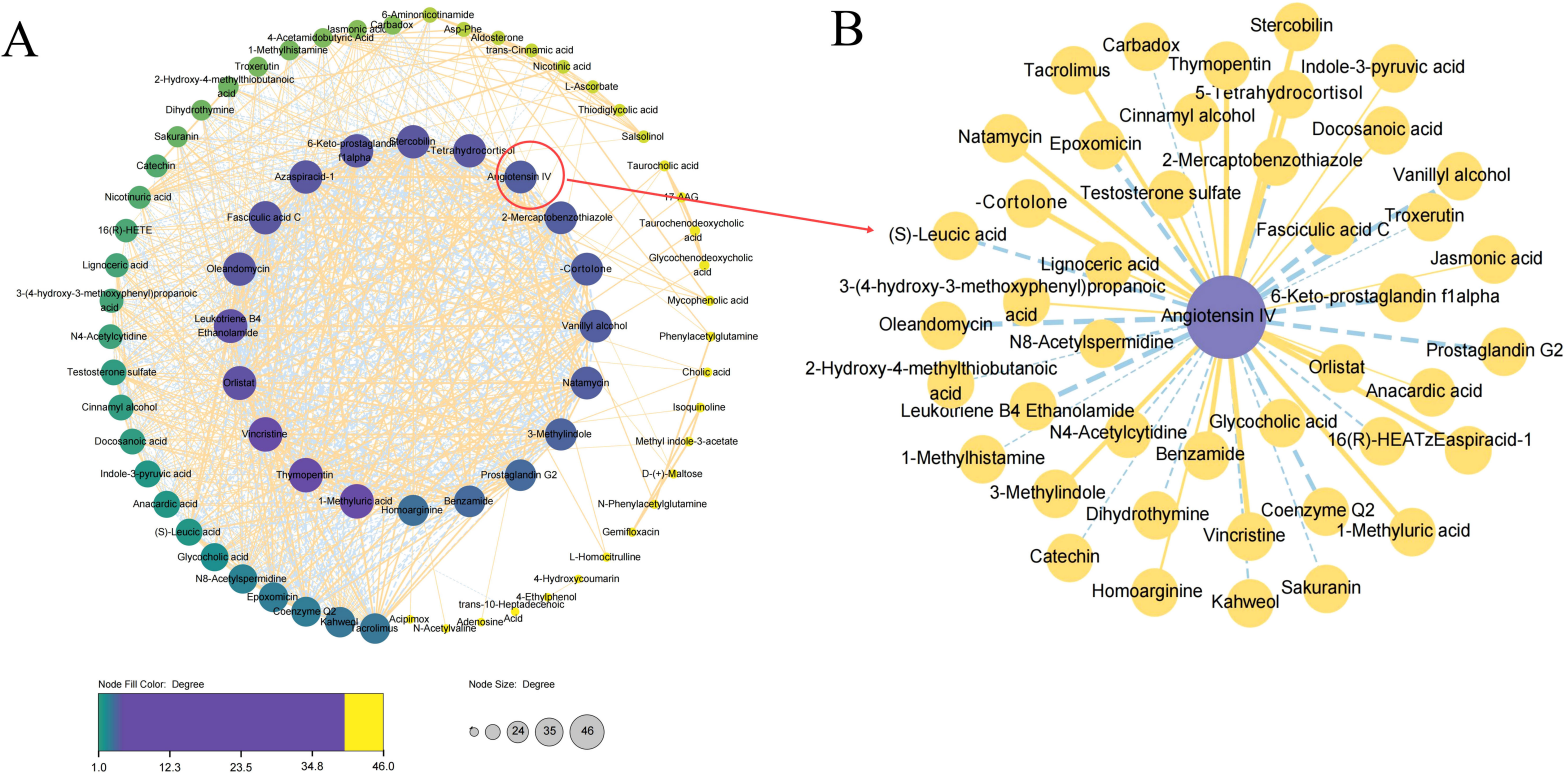
Differential correlation analyses of plasma metabolites in TB patients relative to HC. **(A)** Correlation analyses of all the identified metabolites between TB patients and HCs. **(B)** Correlation analyses of Angiotensin IV.

To decipher the relationship between metabolites, we explored pathologically relevant lipid modules in TB patients relative to HCs. Only differential correlations with empirical P < 0.05 were displayed (**Figure 7A**). The modules of Angiotensin IV in the global network were circled and enlarged (**Figure 7B**).

## Discussion

The identification of new biomarkers for active TB is essential for effectively controlling the ongoing TB pandemic. In China, the Xinjiang Uygur Autonomous Region, which has a high TB burden, reports patient numbers and morbidity rates significantly higher than the national average. Establishing and evaluating novel methods in this region is necessary to provide useful tools for TB management. In this study, we investigated metabolic dysregulation in the plasma of TB patients and identified several key differential metabolites with promising diagnostic potential for TB. Seven core differential metabolites were identified by LASSO, Random Forest, and XGBoost. Among them, Angiotensin IV showed the highest diagnostic value for TB. Our findings provide novel insights into valuable circulating biomarkers and the underlying mechanisms of metabolic perturbations in the pathophysiological processes of TB.

Alterations in the systemic metabolite profile provide a near-holistic view of complex responses and may help characterize the systemic dimension of host-pathogen interactions (Chu et al., 2021). The difficulty of obtaining respiratory tract specimens and the risk of aerosol transmission during sample collection pose challenges to TB diagnosis. The detection of metabolites in plasma provides several advantages for TB diagnosis, including the ability to capture systemic metabolomic changes induced by infections and to investigate metabolic alterations associated with both pulmonary and extrapulmonary TB. It has been reported that metabolites can regulate immune-inflammatory alterations that affect the progression of TB (Ferdosnejad et al., 2024). From the initial stages of MTB infection to the progression of active disease, a range of metabolic pathways induced by invasive bacteria disrupt the host’s immune system, resulting in persistent infection (Weiner et al., 2020). The present study identified a significant percentage of metabolites enriched in lipid metabolism among TB patients. The alterations in metabolic pathways reflected changes in the biochemistry of host cells after MTB infection. Our previous work in childhood TB also revealed a distinct lipid metabolic signature (Sun et al., 2023). Host lipids serve as a primary nutritional source for the growth and reproduction of MTB *in vivo* and comprise the major biomolecules that constitute total cell biomass, acting as the foundation of the eukaryotic membrane (Conover et al., 2008). Amino acids are integral components of all metabolic cycles and are therefore essential for all life forms. The relationship between host amino acids and TB progression has been elucidated. For instance, levels of tryptophan and its downstream metabolites in cerebrospinal fluid have been shown to determine outcomes in tuberculous meningitis (Ardiansyah et al., 2023). A seryl-leucine glycopeptide detected in urine has been demonstrated to be a valuable biomarker for effective anti-TB therapy (Fitzgerald et al., 2019). Nevertheless, there is limited understanding of circulating metabolites that regulate inflammatory responses in individuals with latent TB infection or active TB disease.

In recent years, the development of metabolomics has significantly advanced the identification of new TB biomarkers, shedding light on novel disease mechanisms and enhancing TB diagnostics (Preez et al., 2017). This study identified seven key metabolites responsible for discriminating TB via machine learning algorithms, with Angiotensin IV showing the highest accuracy. Angiotensin IV is a major metabolite of angiotensin II which has been reported to be significantly elevated in patients infected with SARS-CoV-2 (Vaduganathan et al., 2020; Wu et al., 2020). It has been confirmed that Angiotensin IV binds to the angiotensin type-4 receptor, leading to vasodilation, natriuresis, and nitric oxide release, which triggers oxidative stress and inflammation (Cure et al., 2020). Oxidative stress is one of the biological mechanisms that the host has evolved to counteract MTB infection. In contrast to SARS-CoV-2 infection, we detected a low level of Angiotensin IV in active TB patients for the first time. The results of this study may suggest the potential pathogenesis mechanisms through which MTB inhibits oxidative stress by modulating angiotensin IV. However, the underlying mechanisms of Angiotensin IV in TB progression remain to be further investigated. Currently, there is no data available regarding the diagnostic value of Angiotensin IV in infectious diseases. Our results showed that this metabolite demonstrated good diagnostic value in active TB, with a sensitivity and specificity of 98.6% and 100.0%, respectively. Nevertheless, the potential of Angiotensin IV to function as a TB-specific diagnostic biomarker has yet to be fully elucidated. Notably, we included completely healthy individuals as the control group in this study, which may lead to an overestimation of the sensitivity and specificity of the biomarkers. Future large-sample studies should include controls from other infectious diseases to validate the diagnostic efficacy of these biomarkers.

The identification of metabolomic signatures can provide useful information for the early management of TB. A previous study indicated that serum metabolic biomarker panels could be used for TB diagnosis and phenotyping in rifampicin-resistant and sensitive TB cases (Liu et al., 2024). Moreover, another study also found that the prognostic metabolic signatures could predict the development of subclinical disease before the manifestation of active TB (Weiner et al., 2018). Besides metabolites, various host-based biomarkers have been identified by using unbiased omics approaches, including cytokines, antibodies, RNAs, other proteins, combined multiple biomarkers, etc. Several reviews have summarized the implications of these biomarkers in the diagnosis of infectious diseases (Atallah and Mansour, 2022; Nogueira et al., 2022). Additionally, these biomarkers have facilitated the rapid development of point-of-care tests from easily accessible specimens. Nevertheless, there remains much knowledge to be gained in this field. Systematic evaluation research is needed in at-risk populations, such as children, the elderly, and individuals with severe TB or HIV co-infection.

This study used metabolomics and machine learning algorithms (LASSO, random forest, and XGBoost machine) to identify differential metabolites in active TB patients. The use of machine learning algorithms enhances diagnostic efficiency and opens new possibilities for simplifying the comprehensive bioinformatics analyses of metabolites in “big” data. Identifying reliable biomarkers for accurate TB diagnosis will facilitate strategies for disease prevention and early treatment, effectively halting the progression to advanced disease pathology and transmission.

This study has several limitations. First, TB is a highly heterogeneous disease, and the metabolic characteristics of different patients may be influenced by various factors, such as age, gender, genetic background, and co-morbidities. Second, due to the limited sample size, the metabolic markers identified may not be universally applicable to all TB patients. Future large-scale multicenter studies are needed to validate the diagnostic performance of these metabolites across diverse populations. Third, the association of the differential metabolites with clinical phenotypes was not explored in this study. Plasma metabolic levels may serve as markers reflecting clinical conditions such as systemic inflammation and symptom severity. Future studies are warranted to investigate the clinical relevance of the dysregulation of these differential metabolites. Fourth, because of the limited plasma we collected in the study, only metabolites were analyzed. According to the central dogma of molecular biology (Crick, 1970), DNA (genes) are transcribed to mRNA (transcripts) which are translated to proteins, and their activities result in the formation of small molecules (metabolites). As the ultimate downstream pool of proteins, the metabolome can reflect changes in the biochemistry of living cells or organisms more directly, when compared with genetics and proteomics. However, protein-metabolite crosstalk analysis will help us to reveal the potential mechanism of metabolic disorder involvement in active TB. An integrative analysis of multi-omics is needed to reveal the landscape of TB.

In summary, this study identified a series of potential diagnostic biomarkers for TB through metabolomics analysis, demonstrating high diagnostic accuracy. Although further research and validation are needed, these metabolites may provide new ideas and possibilities for TB diagnosis.

## Data Availability

The datasets presented in this study can be found in online repositories. The names of the repository/repositories and accession number(s) can be found in the article/**Supplementary Material**.
