# Supplementary material for: Metabolomics and lipidomics of plasma biomarkers for tuberculosis diagnostics using UHPLC-HRMS
Source: Front Cell Infect Microbiol. 2025 Jun 30;15:1526740. doi: 10.3389/fcimb.2025.1526740 (PMC12256455; doi:10.3389/fcimb.2025.1526740)
Supplement: Supplementary file 1 [file Table1.docx]

**Data availability statement**

The datasets generated and analyzed during the current study are available in the NGDC repository, accession number: PRJCA041719.

**Supplementary Table 1 Details of the differential lipid metabolites between the active TB and healthy controls**

| Metabolites | Formula | Molecular  Weight | TB vs. HC | | | Potential biological roles | Previous reported relevance to TB and other diseases |
| --- | --- | --- | --- | --- | --- | --- | --- |
|  |  |  | *P* Value  （training set） | *P* Value  （testing set） | Trend |  |  |
| Angiotensin IV | [C40 H54 N8 O8](https://pubchem.ncbi.nlm.nih.gov/#query=C40H54N8O8) | 774.40685 | 3.13e-68 | 6.20e-24 | ↓ | Cognitive Enhancement and Memory Modulation, Antifibrotic Actions | Diabetic cardiomyopathy (Zhang et al., 2021). Alzheimer's disease (Royea et al., 2020) |
| glycochenodeoxycholic acid | [C26 H43 N O5](https://pubchem.ncbi.nlm.nih.gov/#query=C26H43NO5) | 449.31489 | 2.61e-06 | 2.00e-3 | ↑ | Hepatic Cytotoxicity and Apoptosis, Role in Cancer Progression and Therapy Resistance | [Hepatocellular carcinoma (](https://www.ipubmed.cn/Archive/Detail/32756004)Shi et al., 2020) |
| methyl indole-3-acetate | [C11 H11 N O2](https://pubchem.ncbi.nlm.nih.gov/#query=C11H11NO2) | 189.07945 | 2.55e-10 | 7.33e-4 | ↓ | Anti-Inflammatory and Analgesic Effects | Metabolic dysfunction-associated steatotic liver disease (Min et al., 2024) |
| dulcitol | [C6 H14 O6](https://pubchem.ncbi.nlm.nih.gov/#query=C6H14O6) | 182.07875 | 6.47e-05 | 8.43e-3 | ↑ | Anticancer effects across multiple tumor models  A potential biomarker in pediatric ​steroid-sensitive nephrotic syndrome (SSNS) | [Hepatocellular carcinoma](https://www.ipubmed.cn/Archive/Detail/31786318) (Lin et al., 2020) |
| Asp-Phe | [C13 H16 N2 O5](https://pubchem.ncbi.nlm.nih.gov/#query=C13H16N2O5) | 280.10633 | 2.75e-06 | 4.24e-4 | ↑ | Anti-Thrombotic Effects  Anticancer Potential  Peptide stability and signaling pathways | Antineutrophil cytoplasmic antibody -associated vasculitis (Liu et al., 2023) |
| benzamide | [C7 H7 N O](https://pubchem.ncbi.nlm.nih.gov/#query=C7H7NO) | 121.05092 | 3.85e-68 | 2.64e-28 | ↓ | Anticancer Mechanisms and Therapeutic Applications  Neuroprotective and Psychotropic Effects​ | clozapine-associated hypersalivation (Miodownik et al., 2023) |
| carbadox | [C11 H10 N4 O4](https://pubchem.ncbi.nlm.nih.gov/#query=C11H10N4O4) | 262.06863 | 7.03e-15 | 2.07e-4 | ↑ | Mutagenicity and Carcinogenic Potential | Modulate gut microbiota (Looft et al., 2014) |

**References**

Lin, X.L., Li, K., Yang, Z., Chen, B., Zhang, T. (2020). Dulcitol suppresses proliferation and migration of hepatocellular carcinoma via regulating SIRT1/p53 pathway. *Phytomedicine*. 66:153112. doi: 10.1016/j.phymed.2019.153112

Liu S, Xu Q, Wang Y, Lv Y, Liu QQ. (2023). Metabolomics combined with clinical analysis explores metabolic changes and potential serum metabolite biomarkers of antineutrophil cytoplasmic antibody-associated vasculitis with renal impairment. *Peer.J*. 11:e15051. doi: 10.7717/peerj.15051

Looft, T., Allen, H.K., Casey, T.A., Alt, D.P., Stanton, T.B. (2014) Carbadox has both temporary and lasting effects on the swine gut microbiota. *Front. Microbiol*. 5:276. doi:10.3389/fmicb.2014.00276

Min, B.H., Devi, S., Kwon, G.H., Gupta, H., Jeong, J.J., Sharma, S.P., et al. (2024). Gut microbiota-derived indole compounds attenuate metabolic dysfunction-associated steatotic liver disease by improving fat metabolism and inflammation. *Gut microbes.* 16(1):2307568. doi:10.1080/19490976.2024.2307568

Miodownik, C., Kreinin, A., Lerner, P.P., Sokolik, S., Lerner, V. (2023).Treatment of Clozapine-Associated Sialorrhea: The Role of Benzamide Derivatives. *J. Clin. Psychopharmacol.* 43(2):171-177. doi:10.1097/JCP.0000000000001655

Royea, J., Hamel, E. (2020). Brain angiotensin II and angiotensin IV receptors as potential Alzheimer's disease therapeutic targets. *GeroScience.* 42(5):1237-1256. doi:10.1007/s11357-020-00231-y

Shi, C., Yang, J., Hu, L., Liao, B., Qiao, L., Shen, W., et al. (2020). Glycochenodeoxycholic acid induces stemness and chemoresistance via the STAT3 signaling pathway in hepatocellular carcinoma cells. *Aging.* 12(15):15546-15555. doi:10.18632/aging.103751

Zhang, M., Sui, W., Xing, Y., Cheng, J., Cheng, C., Xue, F., et al.(2021). Angiotensin IV attenuates diabetic cardiomyopathy via suppressing FoxO1-induced excessive autophagy, apoptosis and fibrosis. *Theranostics*.11(18):8624-8639. doi:10.7150/thno.48561
